# SLIC-Occ: functional segmentation of occupancy images improves precision of EC_50_ images

**DOI:** 10.1186/s40658-023-00600-4

**Published:** 2023-12-11

**Authors:** Alaaddin Ibrahimy, Jocelyn Hoye, Hao Wu, Bart de Laat, Su Jin Kim, David L. Wilson, Evan D. Morris

**Affiliations:** 1 Department of Biomedical Engineering, Yale University, New Haven, CT USA; 2 Department of Radiology, Yale University, New Haven, CT USA; 3 Department of Biomedical Engineering, Case Western Reserve University, Cleveland, OH USA; 4 Department of Psychiatry, Yale University, New Haven, CT USA; 5 Department of Nuclear Medicine, Seoul National University Budang Hospital, Seongnam, South Korea

**Keywords:** Drug occupancy, Brain imaging, PET simulation, EC_50_ images, Functional clustering, Accuracy and precision

## Abstract

**Background:**

Drug occupancy studies with positron emission tomography imaging are used routinely in early phase drug development trials. Recently, our group introduced the Lassen Plot Filter, an extended version of the standard Lassen plot to estimate voxel-level occupancy images. Occupancy images can be used to create an EC_50_ image by applying an *E*_max_ model at each voxel. Our goal was to apply functional clustering of occupancy images via a clustering algorithm and produce a more precise EC_50_ image while maintaining accuracy.

**Method:**

A digital brain phantom was used to create 10 occupancy images (corresponding to 10 different plasma concentrations of drug) that correspond to a ground truth EC_50_ image containing two bilateral local “hot spots” of high EC_50_ (region-1: 25; region-2: 50; background: 6–10 ng/mL). Maximum occupancy was specified as 0.85. An established noise model was applied to the simulated occupancy images and the images were smoothed. Simple Linear Iterative Clustering, an existing k-means clustering algorithm, was modified to segment a series of occupancy images into *K* clusters (which we call “SLIC-Occ”). EC_50_ images were estimated by nonlinear estimation at each cluster (post SLIC-Occ) and voxel (no clustering). Coefficient of variation images were estimated at each cluster and voxel, respectively. The same process was also applied to human occupancy data produced for a previously published study.

**Results:**

Variability in EC_50_ estimates was reduced by more than 80% in the phantom data after application of SLIC-Occ to occupancy images with only minimal loss of accuracy. A similar, but more modest improvement was achieved in variability when SLIC-Occ was applied to human occupancy images.

**Conclusions:**

Our results suggest that functional segmentation of occupancy images via SLIC-Occ could produce more precise EC_50_ images and improve our ability to identify local “hot spots” of high effective affinity of a drug for its target(s).

**Supplementary Information:**

The online version contains supplementary material available at 10.1186/s40658-023-00600-4.

## Introduction

Positron Emission Tomography (PET) is routinely being used to estimate the required dose for a specific drug through PET occupancy studies [1–9]. Typically, regional occupancy values are used to produce one whole brain estimate of drug affinity (EC_50_) value for a cohort of subjects by fitting the occupancy to an *E*_max_ model [10, 11]. Drug doses are selected to reach a minimum therapeutic level without causing any adverse effects [12]. However, this method cannot detect spatial variation in drug affinity in the brain.

Recently, our group introduced the Lassen Plot Filter (LPF), an extended version of the standard Lassen plot to estimate drug occupancy of the voxel-level [13]. Using baseline and post-drug images of volume of distribution (*V*_*T*_), one can generate an occupancy image for a given drug dose. Multiple drug doses and scan times generate drug occupancy images at different plasma concentrations. Our group produced the first EC_50_ image using voxel-level occupancy images generated by LPF that confirmed the suspected spatial variation in effective affinity of CVL-865 for GABA_A_ receptors in the brain [14, 15]. To maximize our confidence in the observed spatial variation of EC_50_ images, we sought to minimize the variability in the EC_50_ images.

The main goal of this study was to increase the precision of the EC_50_ images while maintaining accuracy. We modified a k-means clustering algorithm, Simple Linear Iterative Clustering (SLIC), to segment 4-dimensional occupancy images into clusters of similar occupancy. Cluster-level occupancy values were then fitted to an *E*_max_ model as per normal and a cluster-level EC_50_ image was generated. We refer to our modified SLIC as SLIC-Occ. To compare the precision of the SLIC-Occ output, we simulated 10 noisy occupancy images corresponding to 10 different plasma concentrations using known EC_50_ values. Estimated EC_50_ images, and coefficient of variation (CV (EC_50_)) images were calculated to assess the value of SLIC-Occ clustering on the accuracy and precision of EC_50_ images.

## Material and methods

### Simulated occupancy images

A digital human brain phantom (image size = 121 × 145 × 121, 1 mm isometric voxels) was used to create occupancy images with regional variation in occupancy, corresponding to different plasma concentrations. The brain phantom data was released under the Creative Commons Attribution-NonCommercial license (CC BY-NC) with no end date. Original MRI scans are from OASIS (https://www.oasis-brains.org/). Labelings were provided by Neuromorphometrics, Inc. (http://Neuromorphometrics.com/) under academic subscription. Concentration–response curves were generated according to an *E*_max_ model to generate 10 ideal occupancy images for the 10 different plasma concentrations of drug.

1 $$\text{Occ}={\text{Occ}}_{\text{max}}* \frac{C}{C+ {\text{EC}}_{50}}$$

In Eq. 1, Occ is the occupancy in every voxel of the occupancy image; EC_*50*_ is the predefined EC_50_ value at every voxel of EC_50_ image; Occ_max_ is the maximum occupancy at every voxel; and *C* is the plasma concentration. The 10 different values of *C* were selected between 3 – 279 ng/mL based on an existing human data set, and Occ_max_ was set to 0.85 [16].

**Fig. 1:**
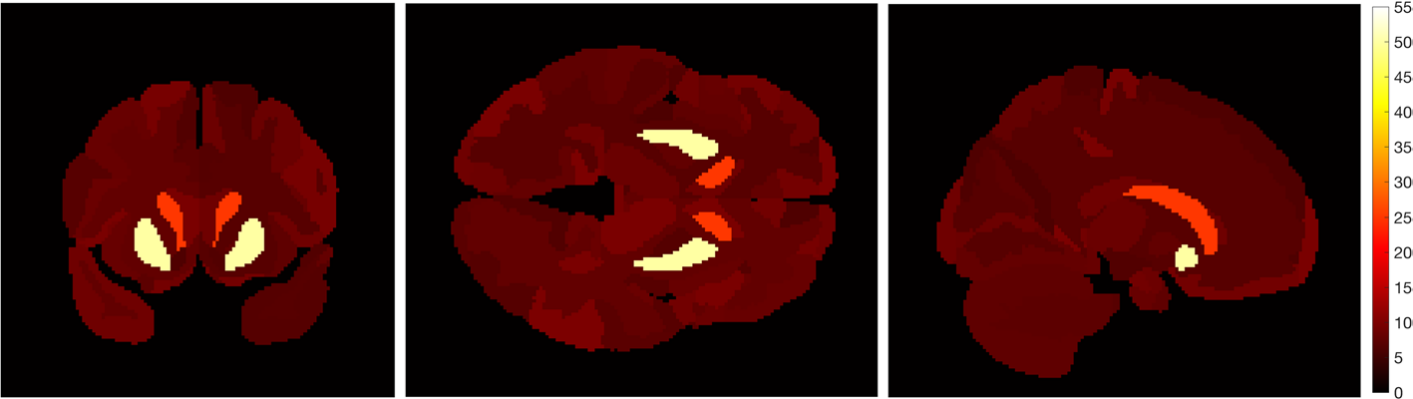
Ground truth EC_50_ image showing “hot spots” of EC_50_ in putamen and caudate regions in coronal, axial, and sagittal views

To generate regional variation in occupancy image, the ground truth EC_50_ image (shown in Fig. 1) was created with an EC_50_ = 25 ng/mL in caudate and EC_50_ = 50 ng/mL in putamen. The rest of the brain was assigned an EC_50_ value between 6 and 10 ng/mL consistent with a whole brain average value from human data shown in a previous study [16].

We have shown previously that noise in occupancy images is a function of occupancy as follows [14].

2 $${\sigma }_{\text{Occ}}=-0.22*\text{Occ}+0.25$$

In Eq. 2, ${\sigma }_{\text{Occ}}$ is the standard deviation in occupancy, and Occ is the occupancy [14]. This noise was applied based on a normal random distribution at every voxel to all idealized occupancy images to the generate noisy occupancy images.

**Fig. 2:**
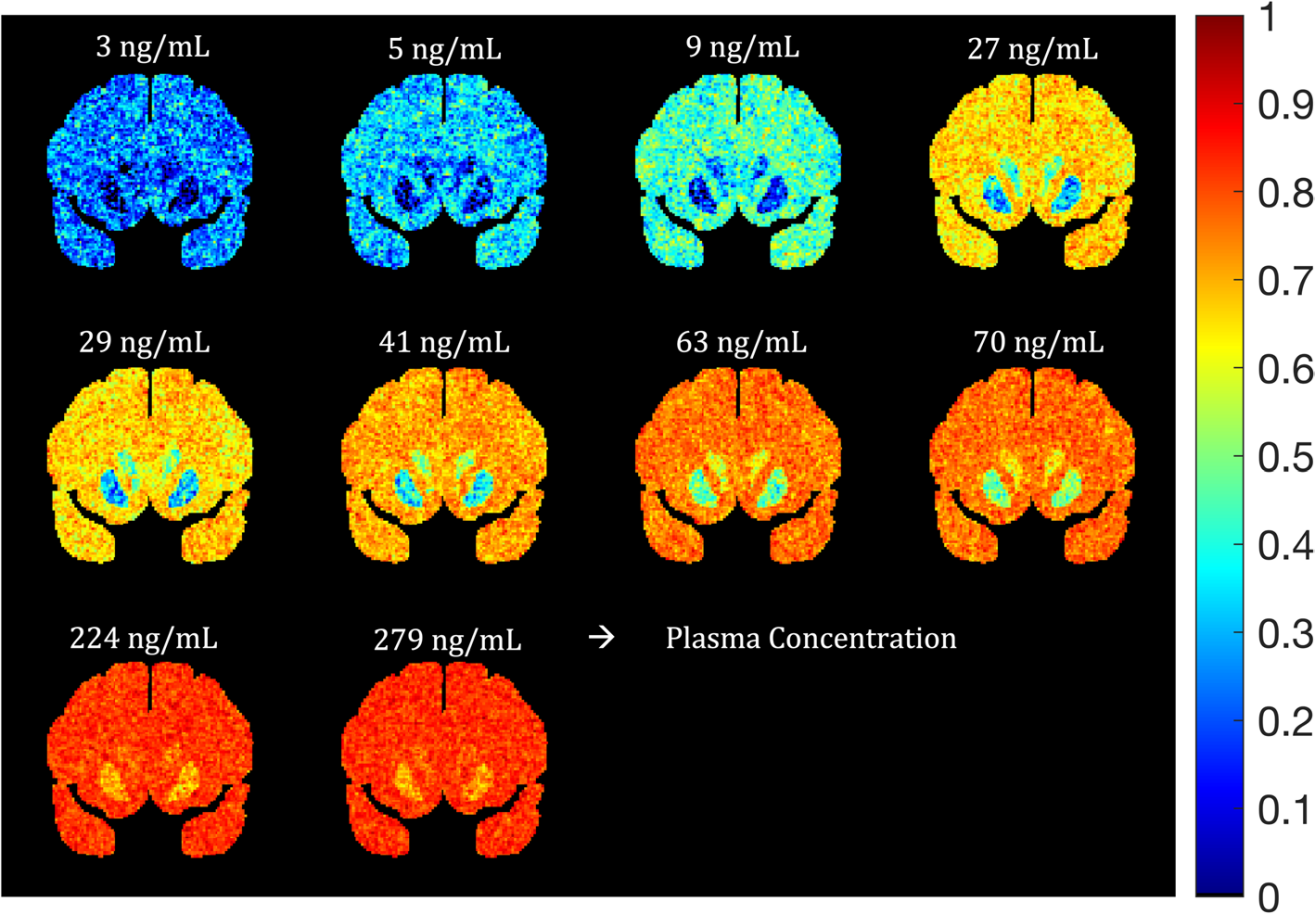
Simulated noisy smoothed occupancy images at different plasma concentrations. The occupancy noise model (Eq. 2) was applied to ideal EC_50_ images (Fig. 1) and smoothed by a Gaussian filter. White text for each image shows the plasma concentration used to generate the occupancy image from the true EC_50_ phantom

To add correlation between the voxels to our noisy occupancy images, we applied a Gaussian filter with a kernel size of 3 × 3 × 3. Multiple phantoms with different amounts of voxel correlation were generated using different Gaussian standard deviations (${\sigma }_{\text{Gauss}}$) for the Gaussian filter. The occupancy images (shown in Fig. 2) generated with ${\sigma }_{\text{Gauss}}$ = 0.5 voxels best represented the smoothness observed in occupancy images produced from real human data [14].

### Clustering

SLIC algorithm was used to combine multiple voxels of the occupancy images into super-voxels (clusters) [17]. SLIC is an adaptation of *k*-means for super-pixel generation. It uses a smaller search area in its distance calculation which is faster than other *k*-means algorithms. It also uses a weighted average of spatial and feature-space distances which can be used to emphasize one of the distances over the other.

#### Mathematical implementation

The SLIC algorithm was first introduced by Achanta et al. [17] for its faster speed, greater memory efficiency and better adherence to boundaries compared to other *k*-means clustering algorithms. Later, a modified version of SLIC, ‘SLICR’, was introduced to incorporate temporal features from 2D dynamic computed tomography myocardial perfusion imaging [18]. In SLIC-Occ, we modified SLIC by introducing a distance in feature-space, feature-space refers to occupancy and spatial distances are calculated in 3D. The total distance measure to be minimized was calculated as:

3 $$D= \sqrt{{d}_{\text{feature}}^{2}+{\left(\frac{{d}_{\text{spatial}}}{S}\right)}^{2}* {m}^{2}}$$

In the Eq. 3, *m* controls the weighting of spatial distance over the feature distance.

4 $${d}_{\text{spatial}}= \sqrt{{\left({x}_{c}- {x}_{i}\right)}^{2}+ {\left({y}_{c}- {y}_{i}\right)}^{2}+ {\left({z}_{c}- {z}_{i}\right)}^{2}}$$

where (*x*_*c*_*, y*_*c*_*, z*_*c*_) is the coordinate of the center of the cluster *c* and (*x*_*i*_*, y*_*i*_*, z*_*i*_) is the coordinate of the voxel *i* which is to be assigned to a cluster. Feature-space is made up of $V$ occupancy images corresponding to different plasma concentrations. Distance in feature-space is:

5 $${d}_{\text{feature}}=\sqrt{{\sum }_{v =1}^{V}{\left({f}_{c}^{v}-{f}_{i}^{v}\right)}^{2}}$$

where, ${f}_{c}^{v}$ is the occupancy value at the center of cluster *c* corresponding to plasma concentration value *v* and, ${f}_{i}^{v}$ is the occupancy value of the voxel *i* corresponding to plasma concentration value *v.* The search area for every voxel was defined as *2S* x *2S* x *2S*, where *S* is defined as:

6 $$S= \sqrt[3]{N/K}$$

In Eq. 6, *N* is the total number voxels in each 3D occupancy image and the image is divided into *K* clusters.

#### Hyper-parameter selection

There are two parameters (i.e., *m* and *K*) that need to be optimized for SLIC-Occ clustering algorithm. The number of initial clusters,* K*, will determine the approximate size of a cluster, *N/K*, in terms of voxels. While the shape of each cluster will be determined by the value of *m*; the larger the *m* the more regular the clusters.

We created multiple simulations with different combinations of *m and K* to investigate their effects on accuracy and precision of EC_50_ images in our clustering algorithm. The choice of *m and K* was made to reduce the CV(EC_50_) while maintaining accuracy of EC_50_.

### EC_50_ estimation

SLIC-Occ was used to segment 10 occupancy images, corresponding to 10 different plasma drug concentrations, into super-voxels (*K* clusters). Average occupancy of all the voxels within each cluster was used as the occupancy value for the corresponding cluster. Two versions of the *E*_max_ model (Eq. 1), were used to fit the occupancy data. In version 1, Occ_max_ was fixed (1-parameter model); in the 2-parameter version Occ_max_ and EC_50_ were estimated simultaneously.

The corrected Akaike information criterion (AICc) was calculated for both (1-parameter and 2-parameter) *E*_max_ model fits at every concentration–response curve (i.e., every cluster) as [14, 19]:

7 $$\text{AICc}=2p+n*\text{ln}\left(\frac{\text{SSE}}{n}\right)+ \frac{2{p}^{2}+2p}{n-p-1}$$

where *p* is the number of estimated parameters in the model, *n* is the number of data points being fitted, and SSE is the sum of squared errors. The model with lower AICc was selected as the best model.

Parametric images were constructed with the parameter estimate of the best model for each cluster. In other words, the final parametric images generated are a combination of 1- and 2-parameter fits depending on which model was selected based on AICc for each cluster. Using the cluster-level best-fit, EC_50_ and CV(EC_50_) images were generated. The coefficient of variation for an estimated parameter was defined as:

8 $$\text{CV}=\frac{{\sigma }_{\text{fit}}}{\mu }$$

where $\mu$ is the parameter estimate, and ${\sigma }_{fit}$ is the standard deviation of the parameter estimate calculated by:

9 $${{\sigma }_{\text{fit}}}^{2}=\text{diag}\left({\left({J}^{\prime}*J\right)}^{-1}*\left(\frac{{R}^{\prime}*R}{n-p}\right)\right)$$

where *J* is the Jacobian matrix of *E*_max_ fit at the solution, *R* is the vector of residuals, *n* is the number of fitted data points, and *p* is the number of estimated parameters.

The distinct image regions in the ideal EC_50_ image were used to select the same voxels in the estimated EC_50_ image for bias calculation. Accuracy was calculated as percent bias as:

10 $$\text{Accuracy}= \frac{\text{Estimated\,EC}_{50 }- \text{Ground\,truth } {\text{EC}}_{50 }}{\text{Ground\,truth } {\text{EC}}_{50 }}*100$$

### Voxel-level fitting of occupancy data

The two versions of the *E*_max_ model (1-parameter and 2-parameter), were used to fit the noisy occupancy data at every voxel. As with cluster-level estimates, voxel-level parametric images were constructed with the parameter estimate of the best model at each voxel, using the AICc to determine the best model fit. EC_50_, and CV(EC_50_) images for voxel-level estimation were generated to compare with the results from cluster-level images.

### Human occupancy

SLIC-Occ algorithm was applied to human occupancy data (image size = 121 × 145 × 121, 1 mm isometric voxels) that were published in previous studies [14, 16]. A detailed description of the PET acquisition has been published [16]. In short, 5 healthy subjects underwent 3 scans each at the Yale PET center for 2 h on a ECAT EXACT HR + scanner (Siemens Medical Systems, Knoxville, TN, USA) after injection with 570 ± 141 MBq (injected mass: 2.7 ± 1.3 µg) of ^11^C-flumazenil, a nonselective GABA_A_ tracer. One of the scans was performed at the baseline (no drug administration), and two were acquired after oral administration of a single acute dose of the $\alpha 1$-, $\alpha 2$-, $\alpha 3$-selective GABA positive allosteric modulator, CVL-865 (also known as PF-06372865). The drug dose was either 10 mg (*n* = 3) or 65 mg (*n* = 2). The post-drug scans were acquired at approximately 1.5 h and 24 h after administration of the drug. The plasma concentration of CVL-865 was measured at three different time points during the scan and averaged [16]. The occupancy versus drug concentration curves were generated for each voxel in the previous study [13, 14]. Using SLIC-Occ, the occupancy images were segmented into clusters. EC_50_, and CV(EC_50_) images were generated and compared with voxel-level EC_50_ and CV(EC_50_) images.

## Results

### Hyper-parameter selection

**Fig. 3:**
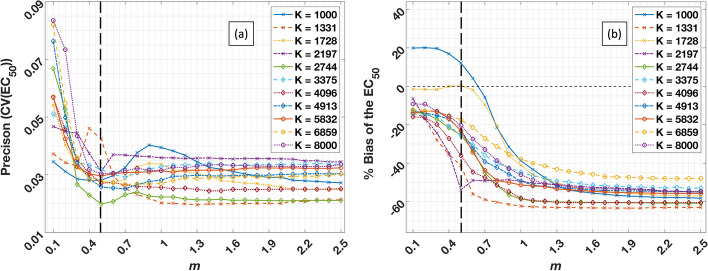
**a** Precision and **b** accuracy (Eq. 10) of EC_50_ for caudate region with true EC_50_ = 25 ng/mL using different *m* and *K* combinations. *K* is the number of initial clusters, and *m* is weighting coefficient spatial distance over the temporal distance. Vertical dash line represents the selected value of m for the reported case. *Note*: *m* and *K* were selected for analysis of both phantom and human data based on similar calculations for multiple regions on the phantom data

We fine-tuned the two hyper-parameters in SLIC-Occ (1. the number clusters: *K*, and 2. the shape parameter: *m*) by analyzing the precision and accuracy of clustering results with different hyper-parameter combinations. Figure 3a shows the CV(EC_50_) of the caudate region for multiple *m* values between 0.1 and 2.5 in increments of 0.1, combined with multiple *K* values ranging between 1000 (10^3^) to 8000 (20^3^) clusters. The CV(EC_50_) decreases as *m* is increased from 0.1 – 0.7 and then plateaus at *m* > 0.8. The effect of choice of *K* parameter on CV(EC_50_) was minimal.

Figure 3b shows the accuracy of clustering results for the same *m* and *K* combinations and same region (i.e., caudate region with ground truth EC_50_ = 25 ng/mL) as in Fig. 3a. Almost all the simulations with any *m* and *K* combinations showed a negative bias except for the simulation with *K* = 1000. The bias increased as *m* increased from 0.1 to 1.3 for all *K* values and at *m* > 1.4 the bias started to plateau in a range 40 – 60% depending on *K*. Based on Fig. 3, we chose an *m* = 0.5 and *K* = 8000 to cluster occupancy images for both digital phantom and human dataset.

### Phantom data

**Fig. 4:**
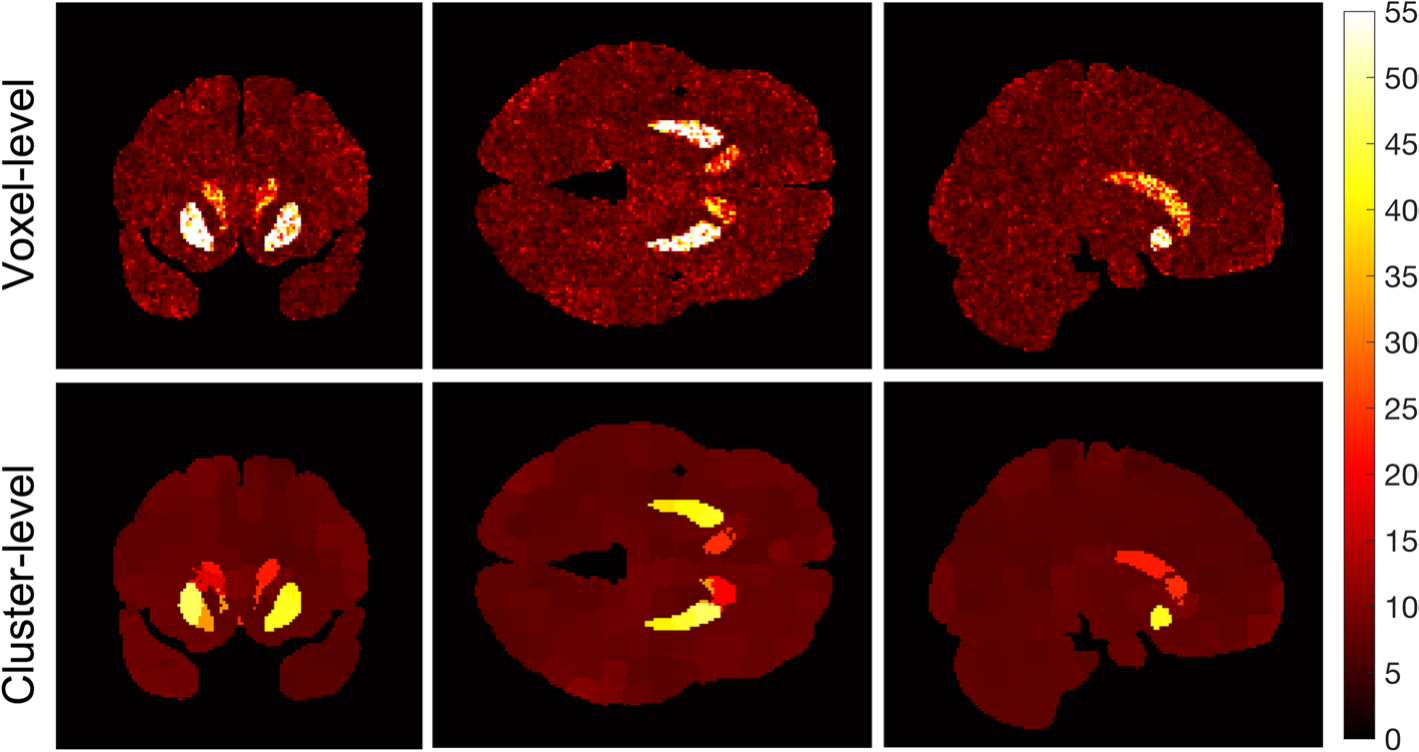
EC_50_ images (ng/mL) for both voxel-level (top row) and cluster-level (bottom row) best-fit (a combination of 1- and 2-parameter models based on AICc selection) shown in coronal, axial and sagittal views

**Fig. 5:**
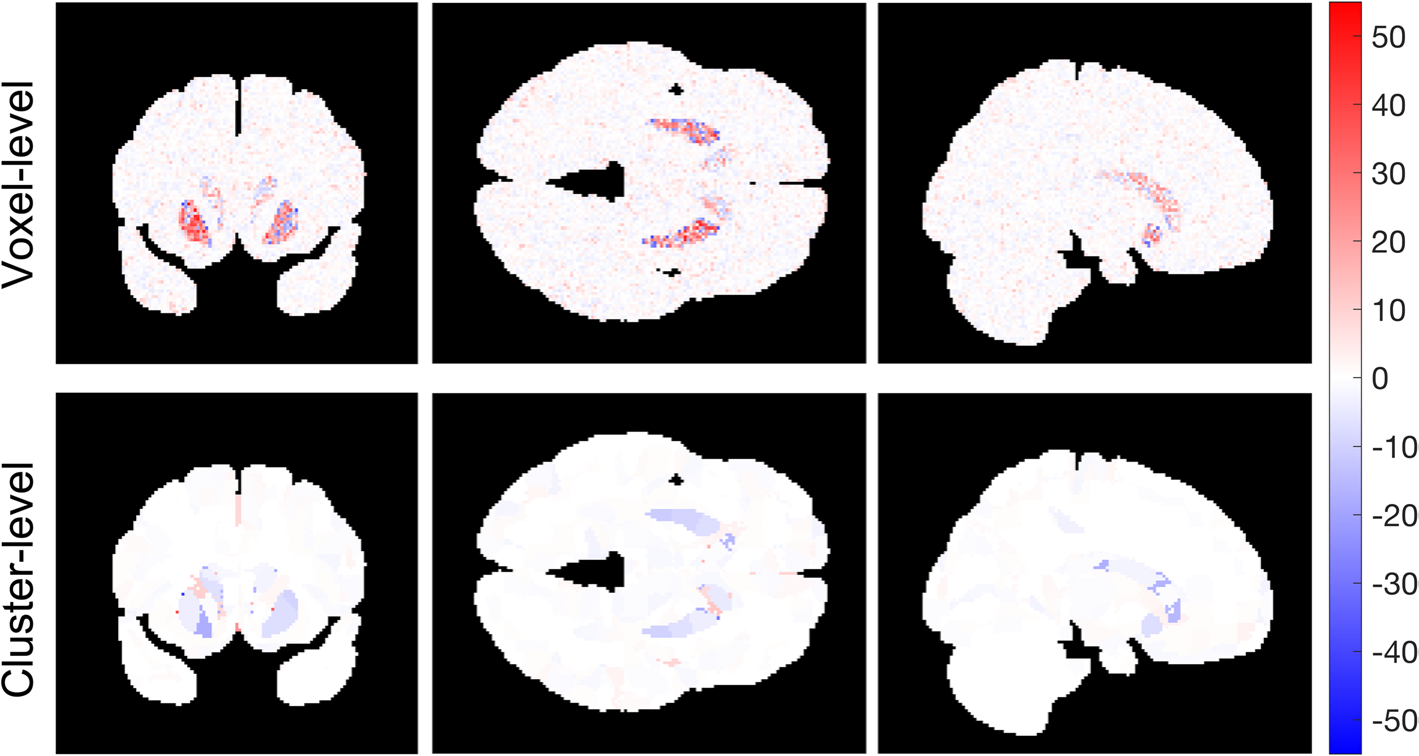
The bias (estimated EC_50_ – true EC_50_) for the voxel-level (top row) and the cluster-level (bottom row) in ng/mL shown in coronal, axial and sagittal views. The voxel-level generally overestimates, while cluster-level underestimates the EC_50_ values in the hot spots

**Fig. 6:**
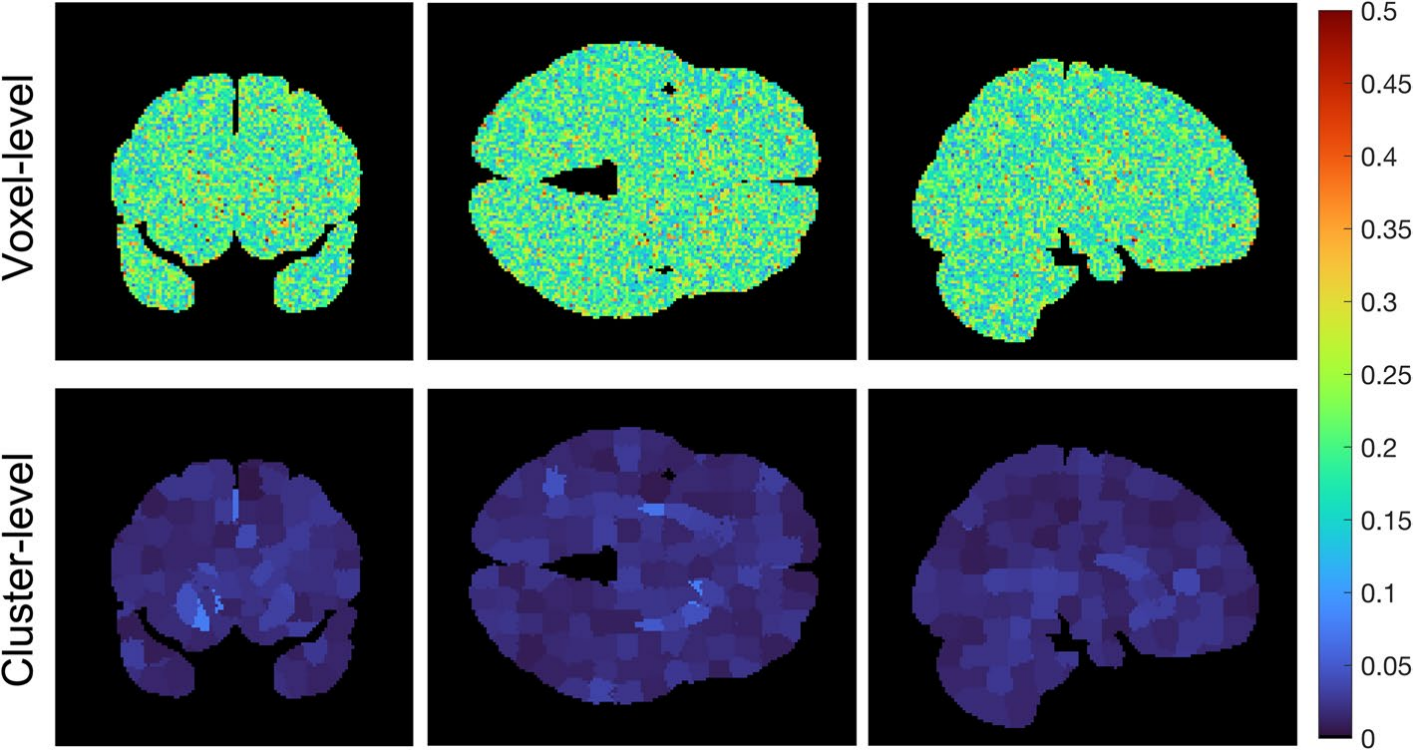
The variability, CV(EC_50_), in voxel-level (top row) is reduced by clustering (bottom-row)

We compared the accuracy, precision, and computational efficiency between voxel-level and the SLIC-Occ parameter estimation using the digital phantom. EC_50_ was underestimated by the cluster-level and was over-estimated by the voxel-level mainly in the hot spot regions (Figs. 4 and 5). However, the precision of EC_50_ was improved using clustering compared to voxel-level (Fig. 6). The 2-parameter model was preferred everywhere when using clustering in occupancy-space, while the 1-parameter model was preferred for a region with high EC_50_ values when using voxel level processing, alone (Additional file 1: Fig. S1). Similarly, the cluster-level analysis, produced Occ_max_ estimates closer to the true Occ_max_ (True Occ_max_ = 0.85) everywhere in the brain than did voxel-level processing. Voxel-level processing tended to overestimate Occ_max_ (Occ_max_ = 1) in the region with the highest EC_50_ (Additional file 1: Fig. S2).

The average EC_50_ and CV(EC_50_) for the putamen, caudate and the whole brain are provided for the ground truth, voxel-level fitting, and cluster-level fitting in Table 1. The CV(EC_50_) in voxel-level was decreased > 5 times by clustering. Clustering decreased the CV(EC_50_) by > 7x, > 5x, and > 10 × in putamen, caudate and whole brain, respectively. Cluster-level computation time was decreased to ~ 6 min compared to 120 min for voxel-by-voxel in voxel-level (Table 1).

**Table 1:** EC_50_ (measured in ng/mL) and CV(EC_50_) are reported as mean ± standard deviation of all the voxels within the region for both phantom and human occupancy data

	EC_50_ (ng/mL)			CV(EC_50_)			Computation time (min)
	Caudate	Putamen	Whole brain	Caudate	Putamen	Whole brain	
*Phantom data*							
Ground truth	25.00 ± 0.00	50.00 ± 0.00	8.13 ± 3.83	–	–	–	
Voxel-level	26.2 ± 10.47	55.25 ± 21.94	8.46 ± 5.27	0.23 ± 0.08	0.22 ± 0.10	0.20 ± 0.06	~ 120^#^
Cluster-level	19.92 ± 5.57	41.28 ± 4.97	8.03 ± 3.08	0.03 ± 0.01	0.04 ± 0.01	0.02 ± 0.01	~ 4–8*
*Human occupancy study data*							
Voxel-level	–	–	10.75 ± 8.48	–	–	0.27 ± 0.27	
Cluster-level	–	–	9.64 ± 6.03	–	–	0.18 ± 0.12	

#Computation time for fitting all the voxels to *E*_max_ model

*Computation time for clustering and fitting all clusters to the *E*_max_ model. Note that, the time varies depending on the number of clusters (i.e., *K*)

### Human data

**Fig. 7:**
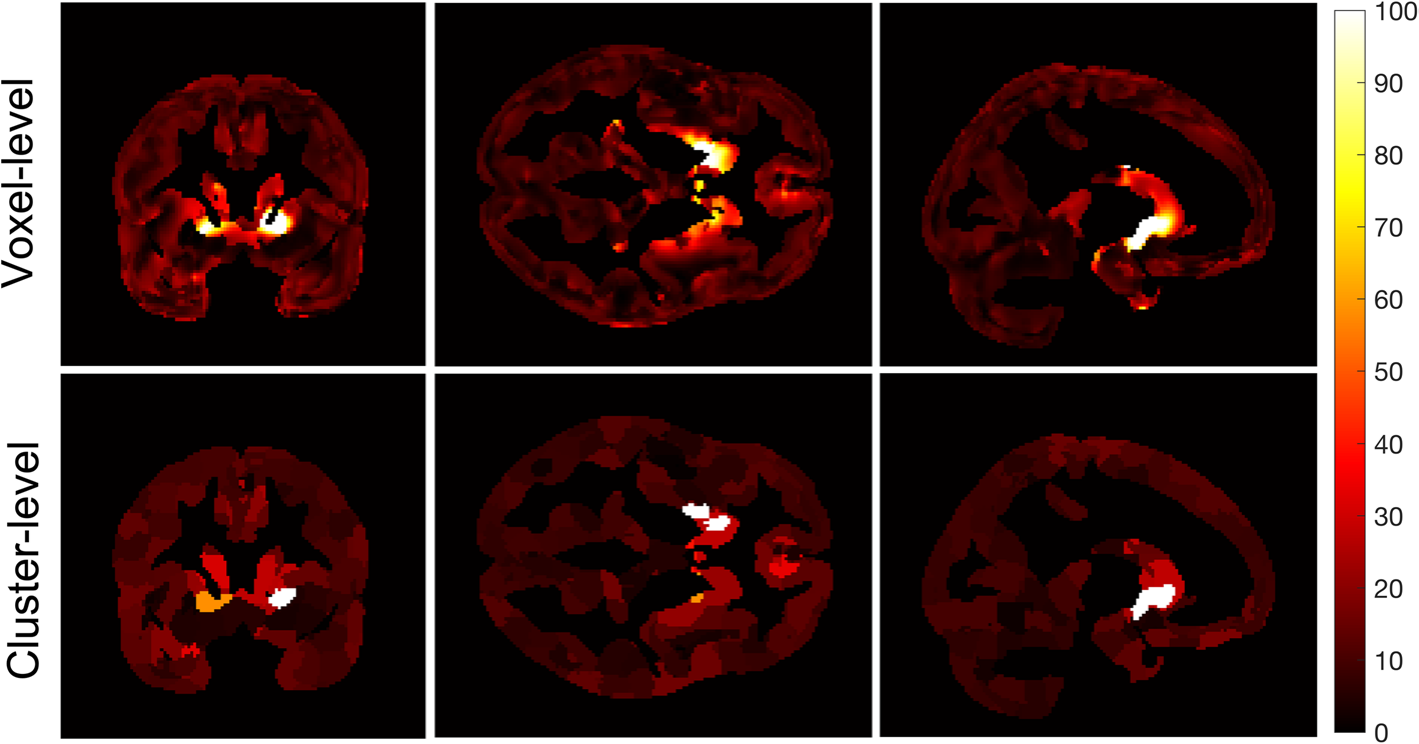
EC_50_ images (ng/mL) for voxel-level (top row) and cluster-level (bottom row) best-fit (a combination of 1- and 2-parameter models based on AICc selection) based on the human occupancy data

**Fig. 8:**
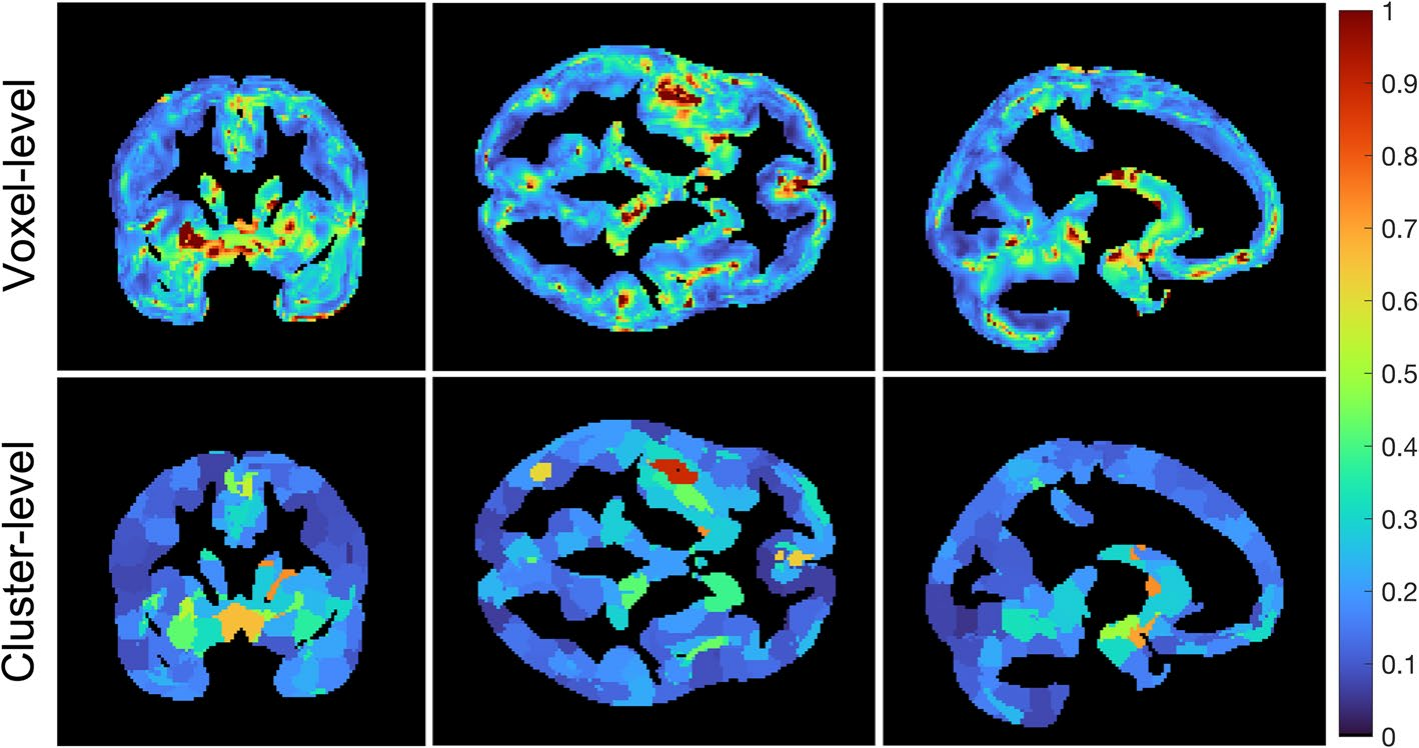
CV(EC_50_) images for voxel-level (top row) and cluster-level (bottom row) based on human occupancy data

Figure 7 shows EC_50_ images for human occupancy data for voxel-level fitting and cluster-level fitting using *m* = 0.5, and *K* = 8000. Regional hot spots can be observed in both methods, but cluster-level fitting produces lower values in the hot spots. This trend is similar to the phantom data, where cluster-level estimated lower EC_50_ compared voxel-level. A lower CV(EC_50_) (i.e., improved precision) is observed for the cluster-level compared to voxel-level (Fig. 8). But the reduction in CV(EC_50_) is not as large as in the phantom data (Fig. 6 and Table 1). The choice of a 1- or 2-parameter model for both voxel-level and cluster-level was less consistent in the human data than in the phantom data (Additional file 1: Fig. S3). However, 2-parameter model was generally preferred over 1-parameter model in the cluster-level compared to voxel-level. Furthermore, the Occ_max_ image followed a similar trend to the AICc image (Additional file 1: Fig. S4).

## Discussion

### Study goal

In this study, we modified the SLIC clustering algorithm and introduced SLIC-Occ to perform functional segmentation of voxel-wise occupancy images into clusters and then to generate an EC_50_ image. Our goal was to create a super-voxel that has the same coordinates spatially, for all the occupancy images, so that we could assign the occupancy data for a cluster to a single binding curve. We expected that by doing so we would reduce the noise in each binding curve, and that would, in turn, reduce variance in the EC_50_ estimates and the computation time.

### Results and their implication

Applying the proposed methodology (clustering using SLIC-Occ) to simulated occupancy images, we estimated the EC_50_ image in the “hot spots” with higher precision compared to the voxel-level method (no clustering), with only a minimal loss of accuracy (Figs. 4, 5 and 6). (Note to reader: The use of “hot spots” refers to high EC_50_, although it corresponds to low affinity in the brain). We observed a similar trend in the human occupancy data, although a smaller improvement in precision was found compared to simulated data (Figs. 7 and 8). The voxel-level method over-estimated the EC_50_ values in the hot spots and the whole brain (compared to ground truth), while the cluster-level method under-estimated the EC_50_ values in the same regions (Table 1). Computation time in the cluster-level method was reduced by orders of magnitude compared to voxel-level (Table 1). This is because there are far fewer binding curves after the occupancy data have been clustered.

Reducing variation in the EC_50_ image was the key goal of our study. Reducing the variance increases the precision of the measurements and increases the power of an occupancy study. This, in turn, can reduce the number of subjects needed to achieve a desired level of statistical power, and thus, can lead to cost savings. Reducing variation, even by a small factor, increases the signal-to-noise ratio of the data, making it easier to detect small differences between groups or treatments. In occupancy images, reduced variance might also aid in the detection of hot spots which differ slightly, but significantly, from the whole brain.

### Reconciling findings in human data and simulations

The CV(EC_50_) in the simulation data was reduced by more than 5 times everywhere, while in the human occupancy data this reduction was less than 2 times. We believe this more modest improvement in the precision could be due to the nature of the variance in human occupancy data that we did not completely replicate in simulation. However, precision for both our simulation and human data were improved using clustering. Further investigation with better models of within-subject and across-subject variations in human occupancy data may shed light on our findings.

### SLIC-Occ implementation

Clustering is becoming popular in many image processing areas. SLIC, a k-means clustering algorithm, was first introduced by Achanta et al. [17] to generate super-pixels in 2D color image more efficiently. Other researchers have modified the algorithm to cluster medical images [18, 20–22]. In our study, we modified SLIC to SLIC-Occ to cluster 4D data (3D occupancy images at different drug concentration levels). The two parameters that must be supplied to the algorithm are *m* and *K*.

We investigated the effects of *m* and *K* on precision and accuracy of the final EC_50_ results by running multiple simulations (Fig. 3, Additional file 1: Fig. S5 and S6). In their study, Wu et al. [18] reported lower average standard deviation in flow (at the voxel-level) with decreasing *m* values. We observed similar results in our simulations: increasing *m* led to increased precision of EC_50_ estimates until it plateaued. Increasing *m* also led to decreased accuracy of EC_50_ estimates. The hyper-parameter, *m* (referred to as a “shape factor” by the Wu et al. [18]), produces more regular hexagonal clusters as it is increased. The effect of increasing *K* was opposite to that of increasing *m*.

In our simulation data, the average CV(EC_50_) using voxel-level fitting was 0.23 for the caudate region (Table 1), which is almost three times as large as the worst CV(EC_50_) predicted by any *m* and *K* combination (Fig. 3a). Selecting an initial large number of clusters (i.e., large *K*) resulted in a smaller bias for the same *m*. Selection of *K* > 4913 (17^3^), and *m* $\epsilon$ [0.3 – 0.5] resulted in a bias between 15 – 20% (Fig. 3b). These combinations of *m* and *K* produced CV(EC_50_) < 0.04, which is smaller than one fifth of the CV(EC_50_) from the voxel-level best-fitting.

In choosing *m* and *K*, one must consider the regularity of the regions that are expected to follow similar behavior as well as the size of those regions. In other words, how small and how ‘regular’ a super-voxel (i.e., a cluster) is needed to capture a region of homogenous behavior, should dictate the choice of *m* and *K*. The larger the *K*, the smaller the super-voxel size and the larger the *m*, the more regular the cluster shapes.

We also checked the reproducibility and directionality of the clustering algorithm by running the same phantom multiple times using different orientations and calculated the EC_50_ at the end of each run. There were no differences between runs suggesting that our algorithm would identify the same clusters every time regardless of object orientation, provided the same *m* and *K* values are entered as inputs. We believe our implementation is not prone to operator bias.

### Limitations

First, the results following clustering depend on selection of the hyper-parameters. In our study, we selected the *m* and *K* values based on multiple simulations and selecting the combination that estimated the lowest precision and the minimum bias. Unfortunately, in real human data the truth is unknown, so the selection of *m* and *K* might require a general understanding of the data, i.e., size, shape and regularity of the clusters. These parameters could also potentially be optimized by further simulation studies.

Second, our simulations may not have perfectly modeled the variability in the human occupancy data throughout the brain. We added noise to our simulated occupancy images at the voxel based on a previously published noise model (Eq. 2). However, the published noise model in Eq. 2 was derived from occupancy in only select regions of the brain and the range of occupancies in these regions may not have spanned the range of occupancies in the whole brain. The simulation data also assumed that all the images were belonged to one ideal binding curve. In effect, we didn’t model inter-subject variability.

Third, the voxel-wise human occupancy data have two main sources of correlation among voxels; (1) the correlation that is generated during the PET scan due to spatial resolution of the scanner, the sensitivity of the detector, and the noise characteristics of the detector, and (2) the correlation that is produced by applying the LPF to V_T_ images. Although, we introduced correlation among voxels in our simulation data by smoothing noisy occupancy images using a Gaussian filter, we believe this filter may not perfectly represent the correlation that is found in the human data. Future studies could potentially address each of the correlation sources separately.

## Conclusion

We introduced a modified version of the SLIC algorithm (“SLIC-Occ”) to segment occupancy images into clusters. Using SLIC-Occ we were able to reduce the CV in the EC_50_ images while introducing minimal bias. Our results suggest that functional segmentation of occupancy images could produce more precise EC_50_ images, improve our ability to identify “hot spots” in EC_50_, and improve power in drug occupancy studies.

### Supplementary Information

**Additional file 1. Figure S1.** AICc images for phantom data showing 1- and 2-parameter selection for both voxel-level and cluster-level in coronal, axial, and sagittal views. **Figure S2**. Occ_max_ images for phantom data showing the maximum occupancy estimated for both voxel-level and cluster-level in coronal, axial, and sagittal views. **Figure S3**. AICc images for human occupancy data showing 1- and 2-parameter selection for both voxel-level and cluster-level in coronal, axial, and sagittal views. **Figure S4**. Occ_max_ images for human occupancy data showing the maximum occupancy estimated for both voxel-level and cluster-level in coronal, axial, and sagittal views. **Figure S5**. **a** Precision and **b** accuracy (Eq. 10) of EC_50_ for a background cluster region with true EC_50_ = 10 ng/mL using different m and K combinations. K is the number of initial clusters, and m is weighting coefficient spatial distance over the temporal distance. Vertical dash line represents the selected value of m for the reported case. Note: m and K were selected for analysis of both phantom and human data based on similar calculations for multiple regions on the phantom data. **Figure S6**. **a** Precision and **b** accuracy (Eq. 10) of EC_50_ for putamen region with true EC_50_ = 50 ng/mL using different m and K combinations. K is the number of initial clusters, and m is weighting coefficient spatial distance over the temporal distance.Vertical dash line represents the selected value of m for the reported case. Note: m and K were selected for analysis of both phantom and human data based on similar calculations for multiple regions on the phantom data.

## Data Availability

Data in this study will be available upon reasonable request.

## Abbreviations

PET: Positron emission tomography
EC_50_: Drug affinity
LPF: Lassen Plot Filter
V_T_: Volume of distribution
SLIC: Simple Linear Iterative Clustering
CV: Coefficient of variation
AIC_c_: Corrected Akaike information criterion
