# Supplementary material for: SLIC-Occ: functional segmentation of occupancy images improves precision of EC50 images
Source: EJNMMI Phys. 2023 Dec 11;10:80. doi: 10.1186/s40658-023-00600-4 (PMC10713928; doi:10.1186/s40658-023-00600-4)
Supplement: Supplementary file 1 — Additional file 1. Figure S1. AICc images for phantom data showing 1- and 2-parameter selection for both voxel-level and cluster-level in coronal, axial, and sagittal views. Figure S2. Occmax images for phantom data showing the maximum occupancy estimated for both voxel-level and cluster-level in coronal, axial, and sagittal views. Figure S3. AICc images for human occupancy data showing 1- and 2-parameter selection for both voxel-level and cluster-level in coronal, axial, and sagittal views. Figure S4. Occmax images for human occupancy data showing the maximum occupancy estimated for both voxel-level and cluster-level in coronal, axial, and sagittal views. Figure S5. a Precision and b accuracy (Eq. 10) of EC50 for a background cluster region with true EC50 = 10 ng/mL using different m and K combinations. K is the number of initial clusters, and m is weighting coefficient spatial distance over the temporal distance. Vertical dash line represents the selected value of m for the reported case. Note: m and K were selected for analysis of both phantom and human data based on similar calculations for multiple regions on the phantom data. Figure S6. a Precision and b accuracy (Eq. 10) of EC50 for putamen region with true EC50 = 50 ng/mL using different m and K combinations. K is the number of initial clusters, and m is weighting coefficient spatial distance over the temporal distance.Vertical dash line represents the selected value of m for the reported case. Note: m and K were selected for analysis of both phantom and human data based on similar calculations for multiple regions on the phantom data. [file 40658_2023_600_MOESM1_ESM.docx]

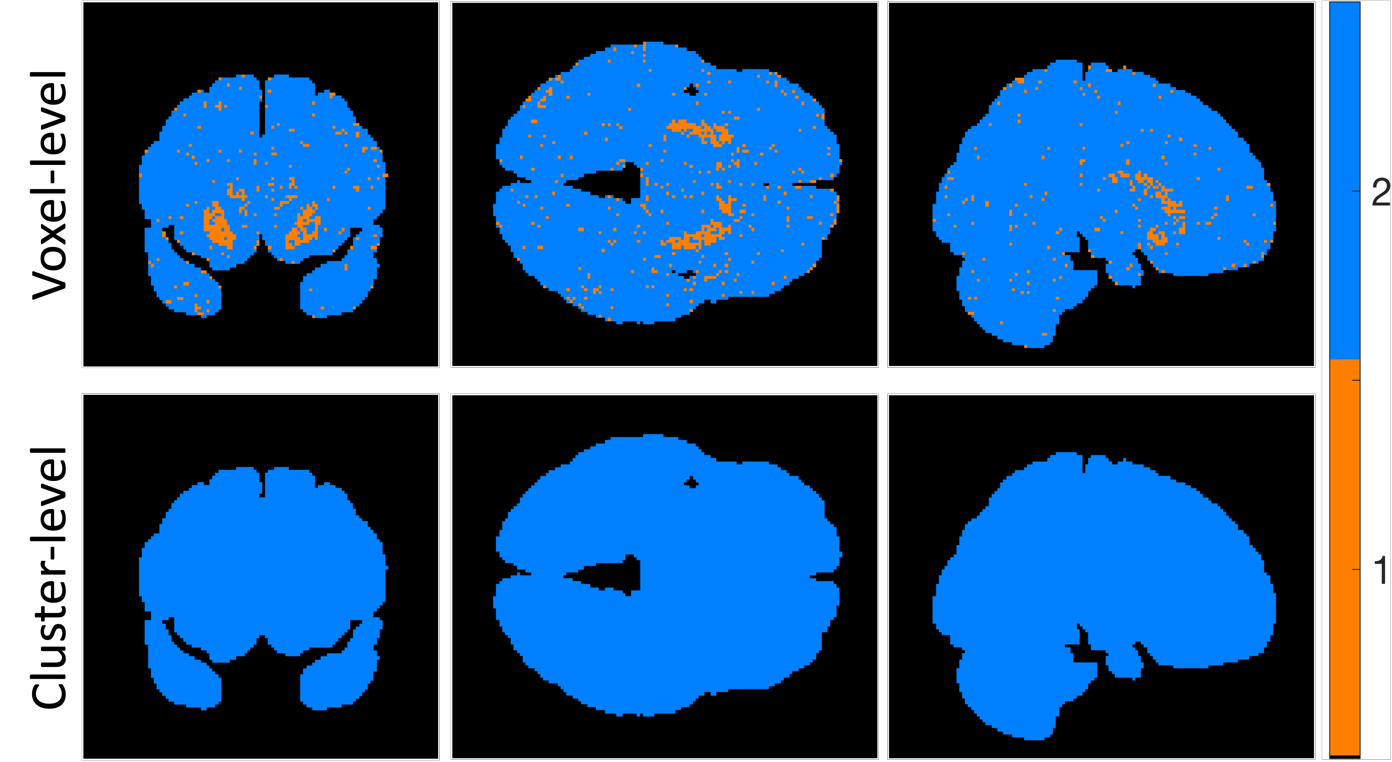


Figure S1 AICc images for phantom data showing 1- and 2-parameter selection for both voxel-level and cluster-level in coronal, axial, and sagittal views.


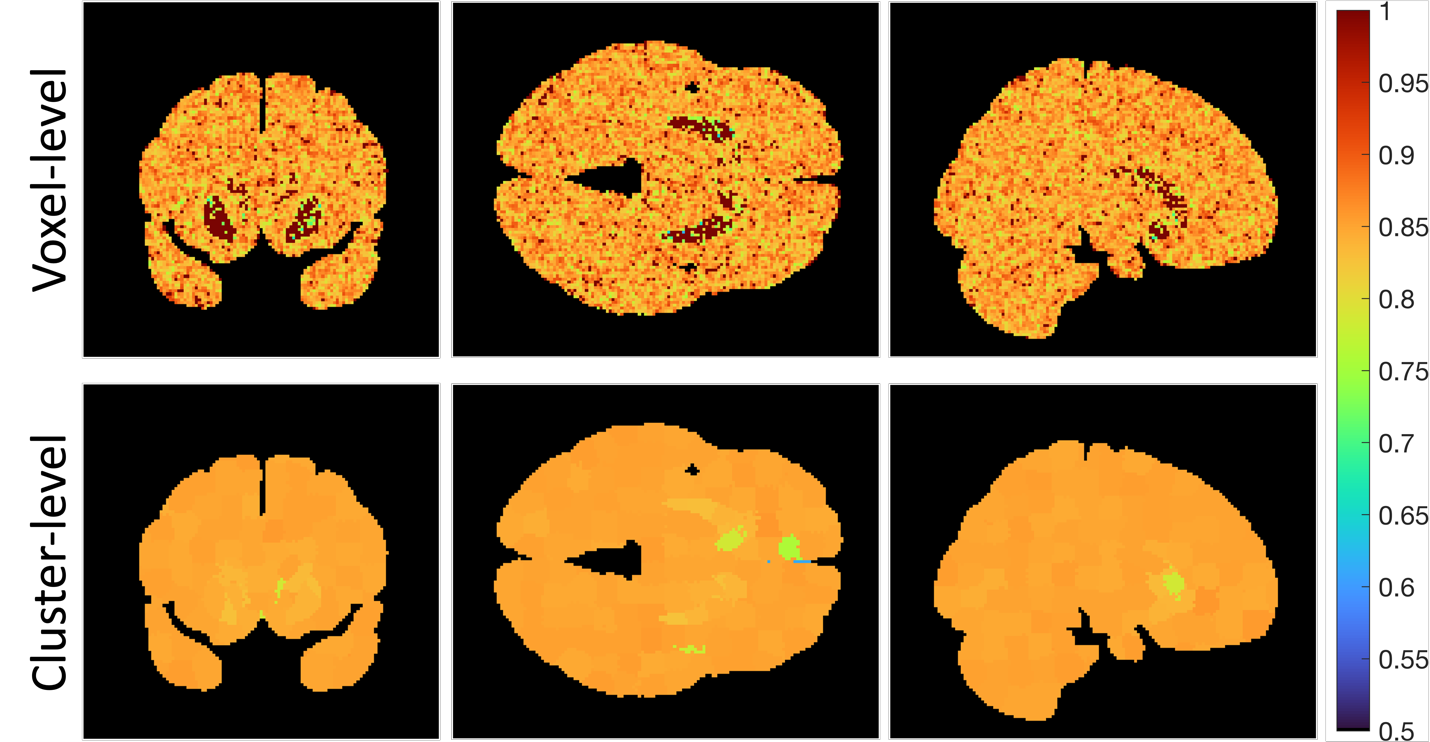


Figure S2 Occ_max_ images for phantom data showing the maximum occupancy estimated for both voxel-level and cluster-level in coronal, axial, and sagittal views.


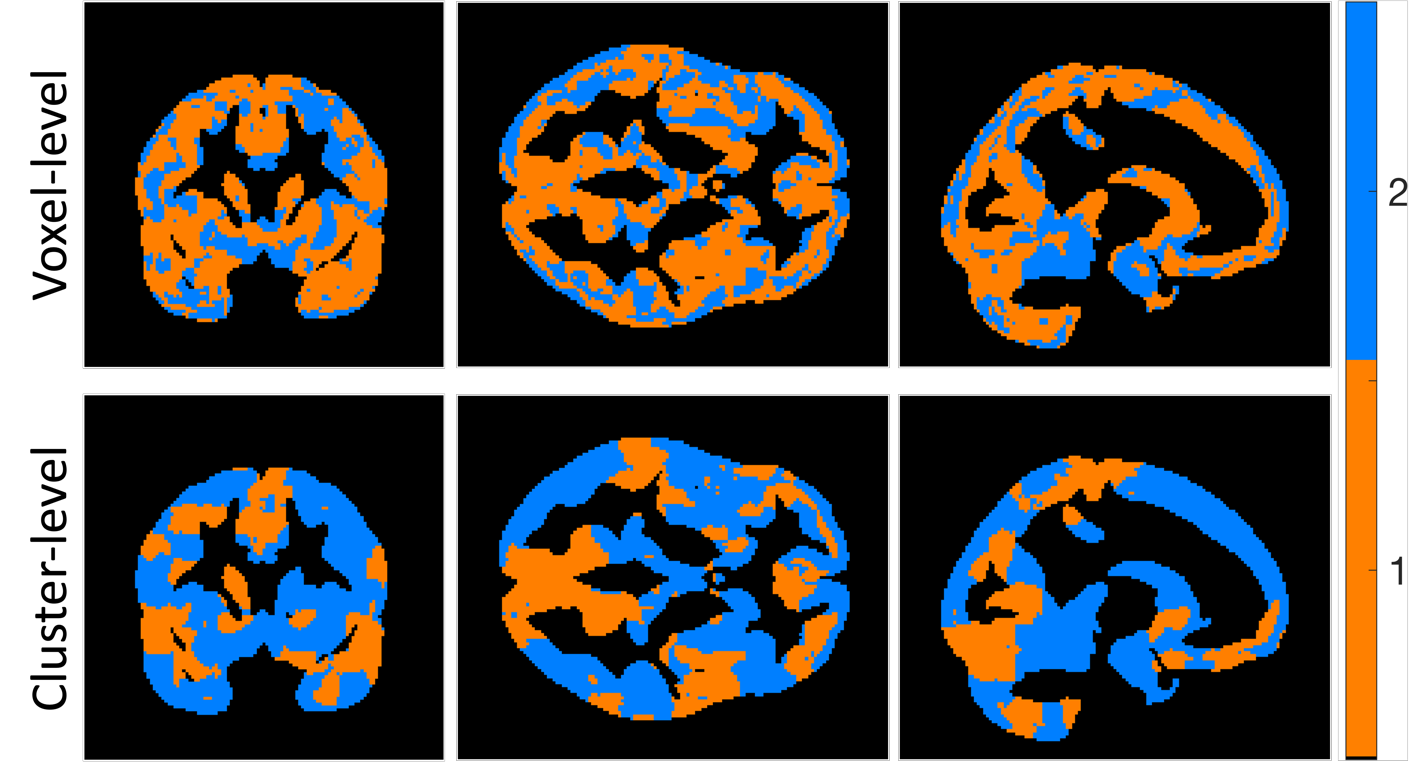


Figure S3 AICc images for human occupancy data showing 1- and 2-parameter selection for both voxel-level and cluster-level in coronal, axial, and sagittal views.


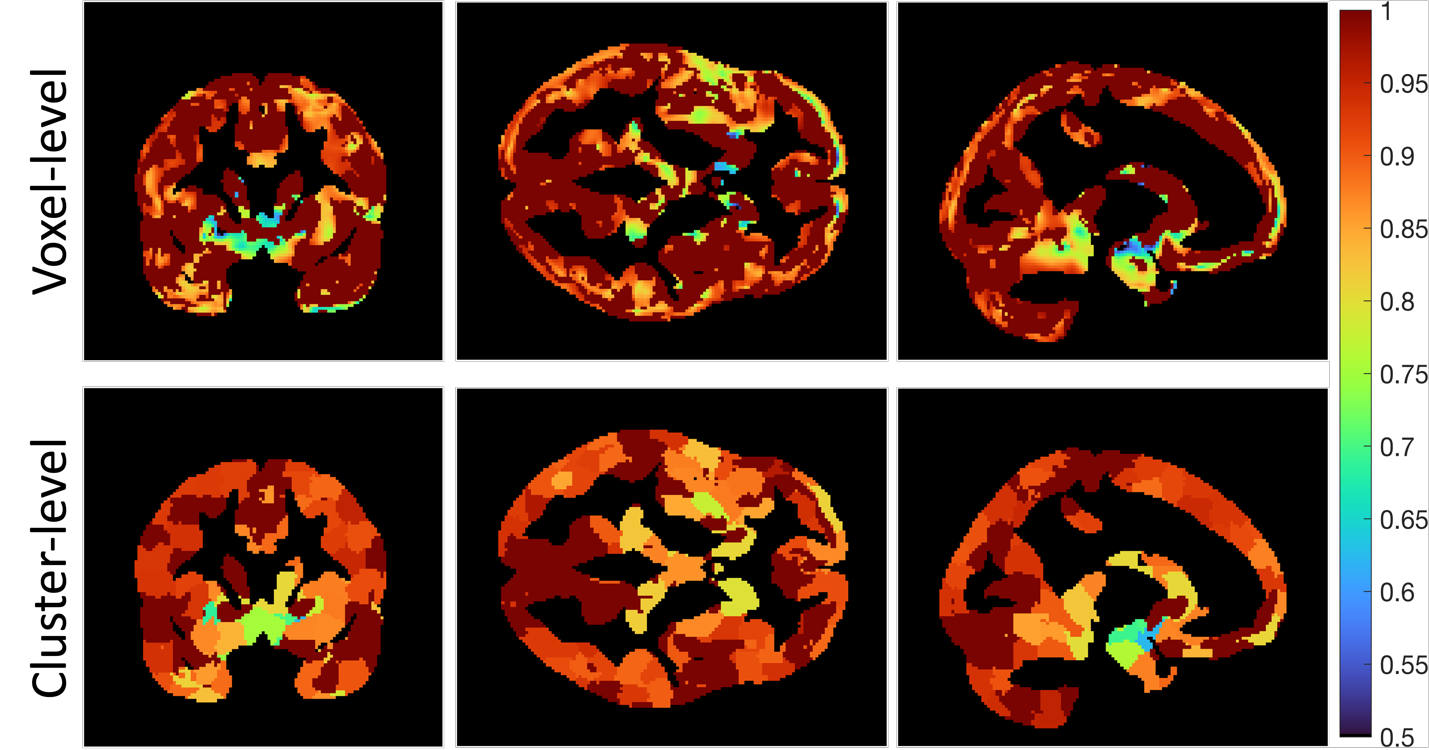


Figure S4 Occ_max_ images for human occupancy data showing the maximum occupancy estimated for both voxel-level and cluster-level in coronal, axial, and sagittal views.


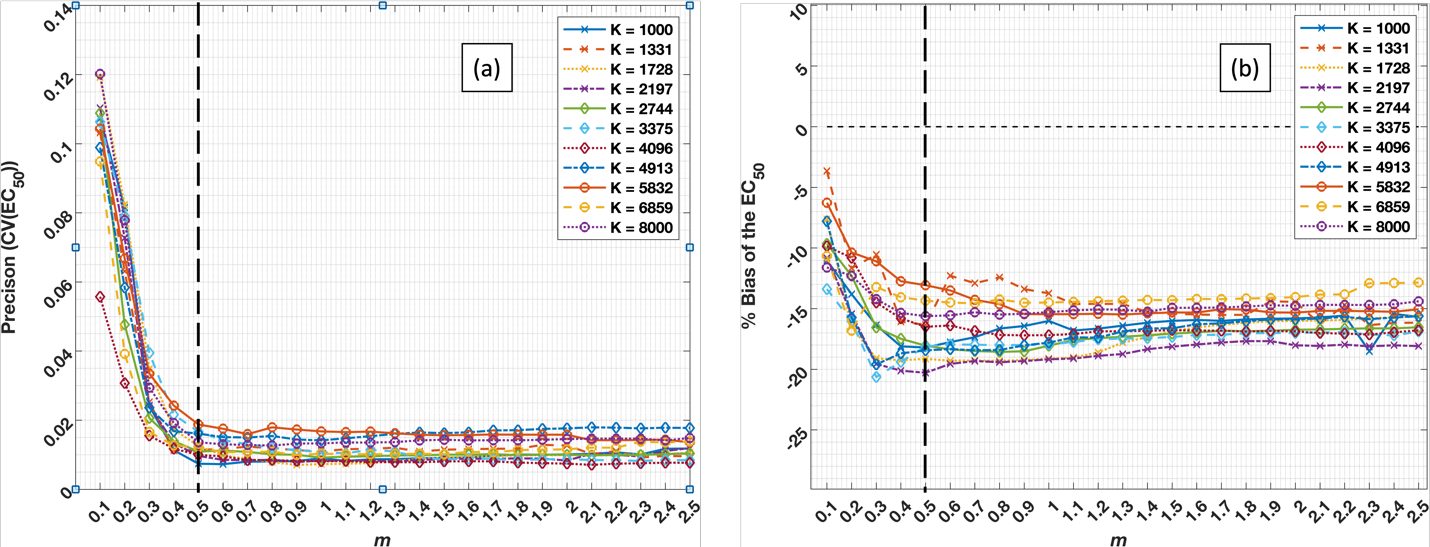


Figure S5 (a) Precision and (b) accuracy (Eq. 10) of EC_50_ for a background cluster region with true EC_50_ = 10 ng/mL using different m and K combinations. K is the number of initial clusters, and m is weighting coefficient spatial distance over the temporal distance. Vertical dash line represents the selected value of m for the reported case. Note: m and K were selected for analysis of both phantom and human data based on similar calculations for multiple regions on the phantom data


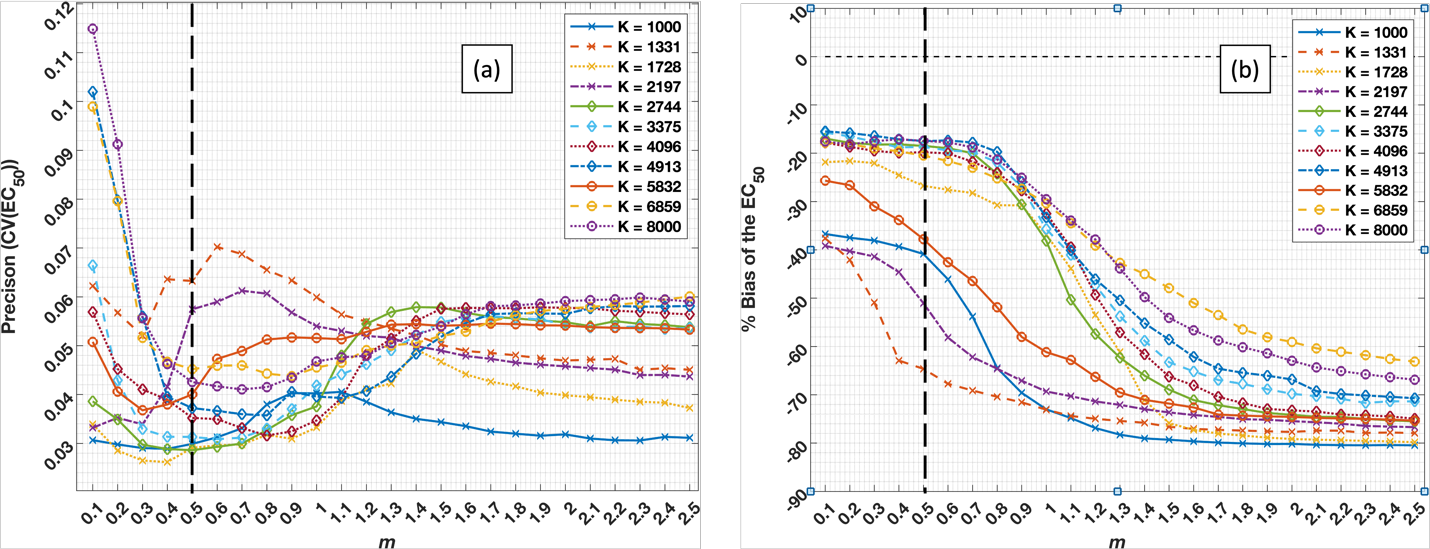


Figure S6 (a) Precision and (b) accuracy (Eq. 10) of EC_50_ for putamen region with true EC_50_ = 50 ng/mL using different m and K combinations. K is the number of initial clusters, and m is weighting coefficient spatial distance over the temporal distance. Vertical dash line represents the selected value of m for the reported case. Note: m and K were selected for analysis of both phantom and human data based on similar calculations for multiple regions on the phantom data
